# Collective Dynamics of Specific Gene Ensembles Crucial for Neutrophil Differentiation: The Existence of Genome Vehicles Revealed

**DOI:** 10.1371/journal.pone.0012116

**Published:** 2010-08-11

**Authors:** Masa Tsuchiya, Vincent Piras, Alessandro Giuliani, Masaru Tomita, Kumar Selvarajoo

**Affiliations:** 1 Institute for Advanced Biosciences, Keio University, Tsuruoka, Yamagata, Japan; 2 School of Media and Governance, Keio University, Fujisawa, Kanagawa, Japan; 3 Environment and Health Department, Istituto Superiore di Sanitá, Rome, Italy; Ajou University, Republic of Korea

## Abstract

Cell fate decision remarkably generates specific cell differentiation path among the multiple possibilities that can arise through the complex interplay of high-dimensional genome activities. The coordinated action of thousands of genes to switch cell fate decision has indicated the existence of stable attractors guiding the process. However, origins of the intracellular mechanisms that create “cellular attractor” still remain unknown. Here, we examined the collective behavior of genome-wide expressions for neutrophil differentiation through two different stimuli, dimethyl sulfoxide (DMSO) and all-trans-retinoic acid (atRA). To overcome the difficulties of dealing with single gene expression noises, we grouped genes into ensembles and analyzed their expression dynamics in correlation space defined by Pearson correlation and mutual information. The standard deviation of correlation distributions of gene ensembles reduces when the ensemble size is increased following the inverse square root law, for both ensembles chosen randomly from whole genome and ranked according to expression variances across time. Choosing the ensemble size of 200 genes, we show the two probability distributions of correlations of randomly selected genes for atRA and DMSO responses overlapped after 48 hours, defining the neutrophil attractor. Next, tracking the ranked ensembles' trajectories, we noticed that only certain, not all, fall into the attractor in a fractal-like manner. The removal of these genome elements from the whole genomes, for both atRA and DMSO responses, destroys the attractor providing evidence for the existence of specific genome elements (named “genome vehicle”) responsible for the neutrophil attractor. Notably, within the genome vehicles, genes with low or moderate expression changes, which are often considered noisy and insignificant, are essential components for the creation of the neutrophil attractor. Further investigations along with our findings might provide a comprehensive mechanistic view of cell fate decision.

## Introduction

Cell fate decision involves reprogramming of precursor cells into the differentiated state. It is intriguing to grasp how a specific path is chosen by a cell, among the several possibilities that can arise, through the complex multi-molecular interactions during differentiation. The understanding of such deterministic process, where the macroscopic stable cell fate transition requires the coordinated regulation of thousands of genes forming networks, could uncover mechanisms that control cell differentiation, as well as reveal better strategy to suppress disease progression, e.g., cancer proliferations.

The study of large-scale network dynamics has been investigated in a variety of fields including mathematics, physics, information sciences, ecology and biology ever since the onset of the nineties [1]–[2]. A large number of studies have already shown that the emergence of collective behavior, such as synchronization of processes, can arise due to the non-linear regulations of complex network systems with environmental perturbations. For example in biology, the secretion and detection of autoinducer molecules between bacteria enable a population of them to collectively regulate gene expression and, therefore, produce coordinated group behavior such as the formation of biofilm by *Pseudomonas aeruginosa* [3]–[4]. However, it remains unclear how the complex and dynamically evolving molecular networks found in biological systems can give rise to a globally coherent orchestrated response.

High-throughput omics (transcriptomics, proteomics & metabolomics) analyses have indicated that the molecular interactions within a living cell typically form a single, largely interconnected network [5]–[8]. It is, thus, necessary to have an integrated network view to understand cellular processes such as cell fate transitions or differentiations in which cells receive a broad range of biological signals or perturbations which influence gene expressions across the entire genome to produce reliable and robust outcome.

To demonstrate the genome-wide integrated response for cell fate decision, Huang *et al.* investigated the differentiation of human pro-myelocytic leukaemia HL-60 cells into neutrophil by the action of two different reagents, DMSO and atRA [9]. Based on the 2773 highest expressed genes (based on two-fold expression changes), Huang *et al.* showed the convergence of cell fate despite different initial transcriptome dynamics arising from the different stimuli, thus suggesting the presence of stable multidimensional attractor states in biology [10]–[13]. Although this result is the first step towards understanding the existence of cell fate attractors, many other fundamental questions remain to be investigated. For example, what are the intracellular origins and mechanisms that instill genome-wide response? What form cellular attractors? How these emerge through the complex molecular networks? If the entire genome is linked through networks, is attractor state achieved by self-regulation [12]?

The majority of large-scale gene expression studies have focused on genes with high expression changes or variations to decipher key regulatory processes, since low-level expression changes of genes have been considered as noisy due to the issue of poor signal-to-noise ratio in microarray experiments. This is due to the difficulty in the estimation of unspecific binding abundance between probe and target in signal intensity [14]–[16], and especially for the low level expression changes, the effect of background noises, compared with specific binding activity, is likely larger than that for highly variable genes. However, in our recent study, we demonstrated that the splitting of whole genome into different ensembles to analyze their temporal expression changes from the initial time resulted in the reduction of their fluctuations as the ensemble size is increased. This resulted in collective genome-wide expression behaviors which exhibited local and global effects of lipopolysaccharide (LPS) stimulated macrophages; *local* being the well-known pro-inflammatory response of a small number of highly expressed genes, while *global* being the novel collective activation of diverse processes comprising the rest of the lowly expressed genes [17]–[18].

In this paper, we investigated the *entire* microarray data of HL-60 cells for atRA and DMSO stimuli including lowly variable signals over time [9]; DMSO is known to activate key transcription factors such as NF-κB [19], whereas atRA penetrates the nucleus and directly remodels chromatin structure [20]. To uncover the orchestrated gene expressions guiding cell fate decision, we used Pearson (linear) correlation and mutual information (nonlinear correlation) metrics to investigate the collective dynamics of gene expressions for each stimulus. To overcome the difficulties of dealing with single gene expression noises in microarray data, we formed grouping of genes (chosen randomly from the whole genome and ranked according to group expression changes across time) which showed the reduction of correlation noises as ensemble size is increased. From this, in contrast to a previous finding which suggested the whole genome's role in differentiation, we demonstrate that only selective portions of fractal-like gene ensembles are responsible for the neutrophil attractor. Notably, the removal of these specific gene ensembles from the whole genome, for both atRA and DMSO stimuli, destroys the attractor. Thus, for the first time, we reveal the existence of ‘genome vehicle’ and show that genes with low or moderate expression changes, contained within genome vehicles, are crucial for the neutrophil attractor.

## Results and Discussion

### Reduction of correlation noises when grouping genes

**Figure 1:**
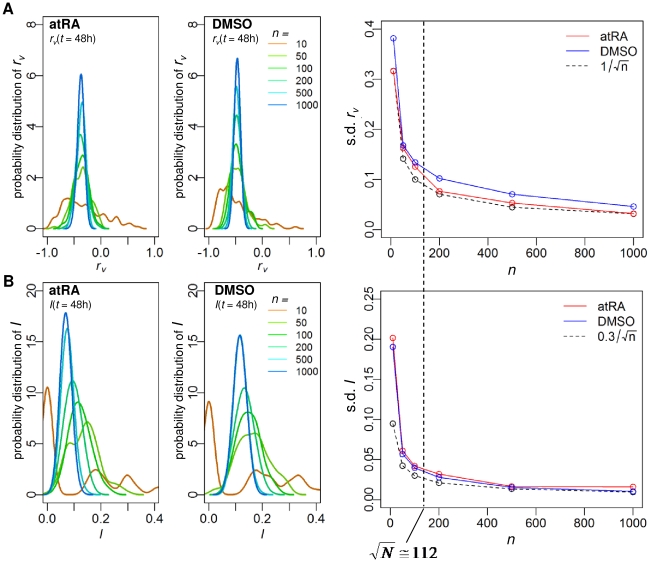
Transition from scattered to smooth bell shaped distributions of *r_v_* and *I* when grouping genes. Distributions of (A) *r_v_* and (B) *I* for ensembles of *n* randomly chosen genes from whole genomes (*n* = 10, 50, 100, 200, 500, 1000), estimated by Gaussian kernel with 100 repeats at a representative *t* = 48h (similar profiles are obtained for all time points), left panels for atRA and middle panels for DMSO response. Standard deviation of *r_v_* and *I* distributions (right panels of (A) and (B)) at *t* = 48h decreases as *n* is increased, following a 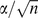 law, *α*≅1 for *r_v_* and *α*≅0.3 for *I*. Note that this transition also occurred for all time points (data not shown).

Previously, we have shown that the collective proinflammatory response of whole genome can be captured by random gene sampling of ensemble size above 80 [17]–[18]. Thus, to investigate the collective behavior of HL-60 cell differentiation, we randomly grouped genes from whole genome into different ensemble sizes (*n* = 10, 50, 100, 200, 500, 1000) and evaluated their expression dynamics in the correlation space (see Methods, “Correlation analysis of gene expressions”). Both the temporal (modified) Pearson correlation of gene variation, *r_v_*, and the corresponding temporal mutual information, *I*, distributions of the gene ensembles transited from scattered and incoherent ones to clear bell-shaped ones for *n_t_*≥100, where 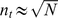 (*N* = 12625, Figure 1). These result show that standard deviations of *r_v_* and *I* distributions at each time point are reduced according to the 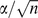 law with increasing *n*, where *α* is the fitting coefficient. Thus, the ensemble size of *n_t_* = 200, with good resolution, was chosen to evaluate the probability distributions of *r_v_* and *I* for each time point of the gene expression data, {***V***(*t_0_*),..,***V***(*t_M_*)}, where ***V***(*t_i_*) is the whole genome expression deviation vector at *t_i_* (*i* = 0,1,..,12) (see Methods).

### Localization and overlapping of probability distributions of correlations for atRA and DMSO responses indicate neutrophil attractor

**Figure 2:**
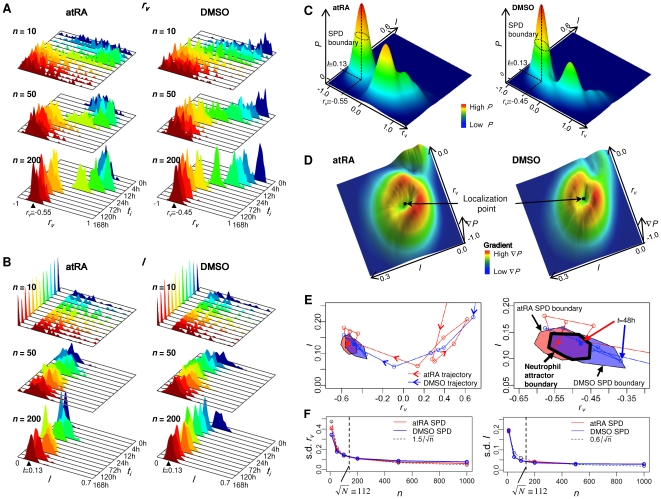
Determination of whole genome attractors for atRA and DMSO responses. Temporal probability distributions of (A) *r_v_* and (B) *I* for atRA (upper panel) and DMSO (lower panel) for *n* = 10, 50, 200 genes randomly selected from whole genome. As *n* is increased, the distributions transit from non-localized to localized at *r_v_*≅−0.55, *I*≅0.13 for *t*≥48h (atRA) and *r_v_*≅−0.45, *I*≅0.13 for *t*≥24h (DMSO), where the bandwidth of Gaussian kernel is given by 0.02 and 0.01 for *r_v_* and *I* distributions, respectively. Note that *r_v_*≅−0.5 represents Pearson *r*>0.94 (Figure S3). Note that to visualize the localization of probability distributions at different time points, intervals are compressed into equal plot intervals. (C) 3D plot of the superposition of the probability (*P*) distributions (SPD) of *r_v_* and *I* over all time points. SPDs were estimated on discretized lattice using the MASS R library (two-dimensional kernel density estimation [34]). The boundary of SPDs for atRA (left panel) and DMSO (right panel), indicated by dotted line, is determined by selecting inflection points on the distributions, where inflection points were estimated on the lattice by selecting the points with highest gradient in 8 adjacent directions from the localization points (peak values of SPDs). Joining these points formed inflection curves for atRA and DMSO responses. (D) 3D plot of the gradient, 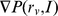, of SPD of *r_v_* and *I* for atRA (left panel) and DMSO (right panel). To obtain the average SPD boundaries (inflection curves), we repeated this process 30 times. Note that *I* was scaled (five folds) to match gradients with *r_v_*. (E) Whole genome trajectories of *r_v_* and *I* for atRA and DMSO are represented by taking the average of 100 trajectories of 200 (*n_t_*) randomly chosen genes from whole genomes for *t* = 0, 2, 4, 8, 12, 18, 24, 48, 72, 96, 120, 144, 168h. Filled polygons indicate the SPD boundaries (inflection curve) for atRA (red) and DMSO (blue). Overlapping of the two SPD boundaries, in purple, indicates the neutrophil attractor. The lines with arrows indicate the whole genome trajectories, red for atRA and blue for DMSO. Dotted curve 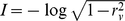 represents the linear correlation of mutual information, *I*, estimated by Gaussian distributions [32]–[33]. Bottom panel: enlargement of the attractor region. The thick line indicates the neutrophil attractor boundary. (F) Standard deviation of the SPDs of *r_v_* (left panel) and *I* (right panel) at the attractor for atRA (red) and DMSO (blue) decreases as *n* is increased, following the 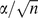 law where *α* = 1.5 for *r_v_* and *α* = 0.6 for *I*.

Utilizing the noise reduction by grouping genes, we plotted the probability distributions of *r_v_* and *I* versus time, and observed that as the ensemble size is increased, the distributions localized to specific points (*r_v_*, *I*), especially after 48h (Figure 2A–B). These localizations may be derived by the presence of neutrophil attractor. To test whether the localization of probability distributions indicate attractor state, we analyzed the superposition of *r_v_* and *I* distributions after 48h for both atRA and DMSO and found they possess distinct peaks for both the atRA and DMSO responses (Figure 2C). Moreover, the superposition of the probability distributions (SPD) of *r_v_* and *I* of atRA and DMSO responses overlap indicating the presence of cell fate attractor, as it corresponds to the fact the two stimuli elicit the same biological end-point, the generation of a mature neutrophil cell.

To define the attractor region, we adopted the concept of critical (inflection) points as used in phase transitions in thermodynamic systems to determine the boundary of the neutrophil attractor. Note that due to the limited temporal data points, we are unable to determine the attractor basin for neutrophil differentiation as defined in continuous dynamics. Thus, we evaluated the gradients of the SPD for *r_v_* and *I* to determine the inflection points for atRA and DMSO responses and the resultant plots reveal distinctive crater-like feature with the rings indicating inflection points (Figure 2D) and the common overlapping area of the inflection points of the SPDs, i.e., the SPD boundaries for the atRA and DMSO responses was defined as the neutrophil attractor (Figure 2E).

As a further test, the attractor boundary also encompasses the convergence of the atRA- and DMSO-trajectories (Figure 2E, right panel). To check the statistical significance of the localized SPD of *r_v_* and *I* within the attractor, we verified that its standard deviation of both *r_v_* and *I* distributions for each stimulus also scales with 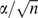 as *n* is increased (Figure 2F). Note that for the other localized SPD of *r_v_* and *I* before 48h, it coincided with the whole genome trajectory loops indicating intermediary cell differentiation states [21] (Figure 2E, left panel).

### Emergence of asymptotic whole genome collective behaviors

**Figure 3:**
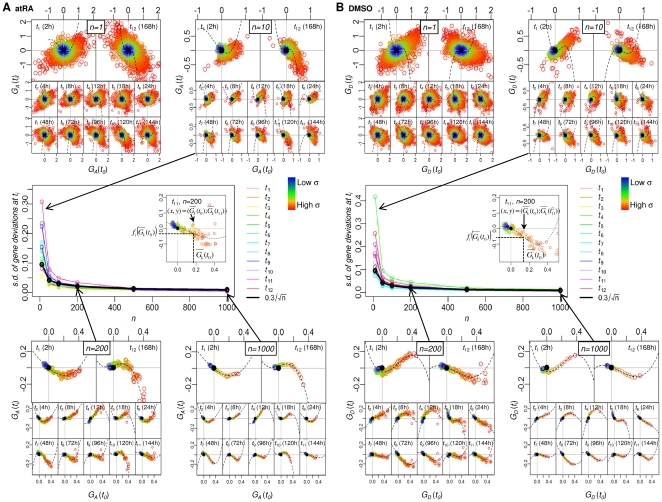
Transition from scatter to asymptotic emergent curves for the ranked groups. Plot of set of mean values of gene deviations for *p* groups, 
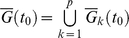
 versus 
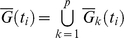
 (*i* = 1,..,12) for atRA and DMSO responses (see maintext and Methods). 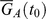 and 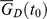 represent mean values of gene deviations for atRA and DMSO respectively. As *n* is increased, the standard deviation of the ranked groups at *t_i_*, 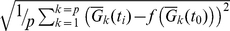, decreases obeying the 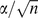 law (center panel, thick black line) where *α*≅0.3, for both atRA and DMSO, with a transition occurring around 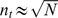 for (A) atRA and (B) DMSO. Each color represents each *t_i_*.

To investigate the whole genome collective behavior, we grouped genes according to their variance across time. The whole genome deviation vector, ***V***(*t_0_*), was sorted from the highest to the lowest standard deviation 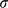 (see Methods, “Ranking gene ensembles”). This sorting order at *t_0_* was retained for all other time points (*t* = 1,..,12). Next, we split the ranked whole genome into *p* groups, where *p* is the integer values of *N/n* for *n* = 10, 50, 100, 200, 500, 1000. Similarly for the random selection of genes, we checked whether expression noises can be reduced for the ranked groups as the group size is increased. We plotted the set of mean values of gene deviations for *p* groups, 
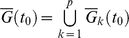
 versus 
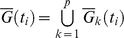
, (*i* = 1,..,12) (see Methods, “Ranking gene ensembles” and Figure 3). The plots show the group's mean values transited from scatter to the emergent asymptotic curves, 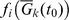, as *n* is increased for all *t_i_* (*i* = 1,..,12) (transition at 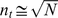, Figure 3A–B). Note that 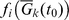 (*k* = 1,2,…,*p*) is the gene deviation value on the asymptotic curve, and *f_i_* is the function of the asymptotic curve for the *i* ^th^ time point determined by the nonlinear least squares fitting with cubic polynomial for the set of points (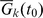, 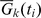). This is due to the fact that as *n* is increased, 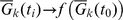, while the standard deviation of the whole genome at *t_i_*, 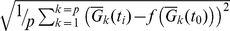, decreases following the 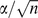 law (Figure 3A–B, center panels).

These asymptotic curves suggest that the genome-wide averaging behavior of collective expression dynamics exists. Once again *n_t_* = 200 genes produced acceptably good resolution. Thus, *n_t_* is the basic size of the genome element, 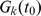, and for the whole genome, we obtained 63 genome elements, totaling 12600 genes. The remaining 25 genes with very low *σ* were discarded. Note: we used *σ* instead of coefficient of variation (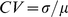) for ranking genome elements as we are dealing with trajectories of ensemble of genes, rather than normalized form as often used in conventional approaches. Nevertheless, we compared *CV* versus *σ* and found linear relationships between them (Figure S1), ruling out any possible trivial scale effect as explanation of our results.

### Specific genome elements fall into the attractor in a fractal-like manner

**Figure 4:**
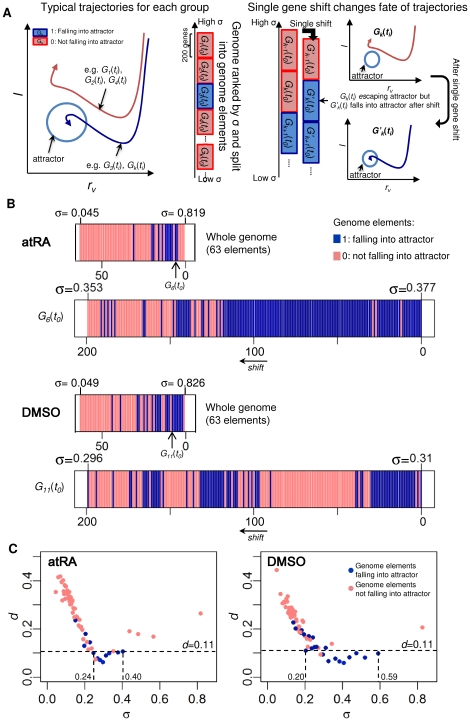
Specific genome elements guiding neutrophil cell fate in fractal manner. (A) Sensitivity of genome elements falling to the attractor against standard deviation: left panel, schematic trajectories of genome elements, 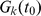 (*k* = 1,…,63), into (blue) and not into attractor (red), sorted by standard deviation *σ*. Right panel, schematic of genome element with single-gene shift can change its fate to the attractor. Illustration shows the process of single-gene shift, i.e., removing the highest *σ* gene from an element 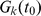 not falling into attractor, and adding the highest *σ* gene of the next lower rank group 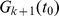, creates a new genome element, 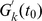, that fall into the attractor. (B) Upper panels: the binary sequence of 63 genome elements atRA and DMSO responses represented by blue and light red, for 1 and 0 respectively. Lower panels, binary sequence of 199 additional genome elements were created using single-gene shift as in (A) for 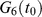 for atRA and 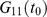 for DMSO. (C) Euclidean distance *d*, between the whole genome's trajectory and each genome element's trajectory. More than 50% of genome elements that fell into the attractor are close to the minimum distance (*d*<0.11, indicated by a dotted line).

We investigated trajectories of the 63 ranked genome elements (Figure 4A, left panel) by creating a sequence of binary numbers where 1 and 0 indicate genome elements falling into and not falling into the attractor, respectively, against their standard deviation, *σ* (Figure 4B, upper panels). The result showed that the genome elements falling into the attractor are non-continuous in *σ*. To understand the discontinuity, we checked the sensitivity of genome elements falling into the attractor, i.e., changing from 0 to 1 or vice-versa for single-gene shift (Figure 4A, right panel). We found that even a single replacement of the highest *σ* gene from a genome element with the highest *σ* gene of the next lower ranked genome element results in its destiny change of falling or not falling into the attractor (see e.g., 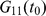 for DMSO, Figure 4B, lower panels). This expansion of a genome element shows fractal-like binary distributions and the sensitivity of single-gene shift within a genome element demonstrates the non-linear nature of gene expression dynamics. Note that the expansion processes are limited by the lack of continuous data to show true fractal characteristics [22]–[23].

Next, we evaluated trajectories of the 63 genome elements and compared each with the whole genome trajectory, in terms of the Euclidean distance, *d*, of *r_v_* and *I*. We observed that 12 genome elements for atRA and 20 for DMSO, fall into the attractor, and among them more than 50% (with 0.24<*σ*<0.40 for atRA and 0.20<*σ*<0.59 for DMSO) are close to the whole genome trajectory with minimum distance (for *d*<0.11) (Figure 4C). This indicates that the genome elements falling into attractor scale with the whole genome trajectory. Overall, these results suggest that whole genome responses possess fractal-like nature for neutrophil differentiation.

To exhaustively search for more possible genome elements that can enter the attractor, we performed an iterative procedure where we removed the initial elements into attractor and shifted the remaining genomes by 50 genes from the highest *σ* values to create new genome elements (Figure S2). Through this, we obtained additional 9 elements for atRA and 8 elements for DMSO.

### The loss of the attractor when specific genome elements are removed

**Figure 5:**
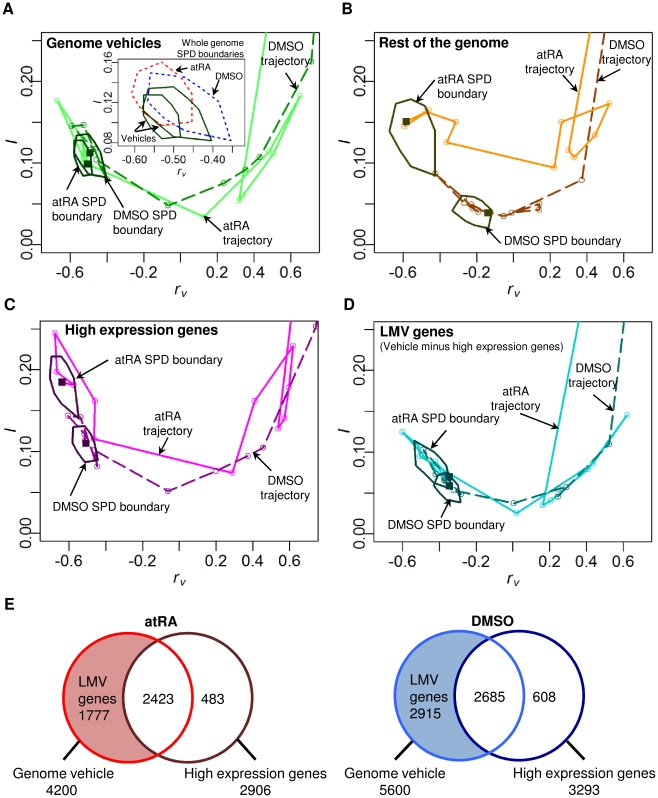
The loss of the attractor when genome vehicles are removed. The SPD boundaries and trajectories for atRA (plain lines with lighter tone) and DMSO (dark dashed lines) responses of (A) genome elements falling into attractor (i.e., genome vehicles) overlap and converge, indicating the formation of neutrophil attractor. (Insert shows overlapping SPD boundaries of atRA and DMSO responses of the whole genomes, indicated by red and blue dotted polygons respectively), (B) rest of genome elements without genome vehicles do not overlap and converge, (C) high expression genes (2-fold change from *t_0_* for at least one time point) of the genome vehicles do not overlap and converge, (D) lowly and moderately variable (LMV) genes of the genome vehicles still overlap and converge, retaining the neutrophil attractor. Data points are represented by circles and last time point by a square. (E) Venn diagrams showing the number of genes constituting the genome vehicles and high expression genes (atRA in red and DMSO in blue). Note that LMV genes constitute 42% and 52% of the genome vehicles for atRA and DMSO responses, respectively.

We evaluated the SPDs of *r_v_* and *I* of all the genome elements into the attractor and found the SPD boundaries of atRA and DMSO responses overlapped to maintained the attractor (Figure 5A), while those for the rest of genome elements did not (Figure 5B). Moreover, for the genome elements falling into attractor, the corresponding trajectories of both atRA and DMSO converged, but not for the trajectories of the rest of genome elements (Figure 5A–B). These results indicate that the rest of genomes for both atRA and DMSO stimuli failed to demonstrate the convergence and to form the neutrophil attractor.

Previously, Huang *et al.* indicated the convergence of atRA and DMSO trajectories in the space spanned by the first two principal components based on 2773 high expression genes (2-fold change in expression values from *t_0_*) [9]. Notably, our analysis shows that for the high expression genes, neither their correlation trajectory converged nor their SPD boundaries overlapped (Figure 5C). Furthermore, SPD boundaries of the specific genome elements without these highly expressed genes maintained a common neutrophil attractor, albeit with less area (Figure 5D). Thus, the lowly and moderately variable genes within genome vehicles play an important role in the formation of the neutrophil attractor (Figure 5E). These results demonstrate that the collective dynamics of specific gene elements for both atRA and DMSO responses are responsible for the cell fate decision and we call these genome elements that effectively drive cells toward the attractor for each stimulus as “genome vehicle”.

In summary, we show the existence of genome vehicles is responsible for neutrophil differentiation. Despite initial differences of the transcriptional program induced by atRA and DMSO stimulations, the self-regulation of the genome vehicles leads to the formation of a common neutrophil attractor. In addition, we demonstrate that the collective motion of lowly and moderately variable genes within the genome vehicle, which are often considered as noisy and insignificant, play an important role in the formation of the neutrophil attractor, perhaps indicating the non-instructive signaling of genes related to small-amplitude DNA motions [24]–[25]. Since the dynamics of gene expression is connected with the dynamics of chromatin structural changes, finding the underlying mechanisms, such as the collective dynamics of small-amplitude DNA fluctuations within chromatin structure, for the motion of the genome vehicle might decipher fluctuations in chromatin dynamics that determines cell fate decision. It will be interesting to know how the concerted motion of the genome vehicle, together with well-known master instructive genes, such as Yamanaka factors [26], drives the differentiation of pluripotent stem cells as well as other biological processes that could acquire a completely different perspective under the proposed model.

## Methods

### Correlation analysis of gene expressions

Microarray technologies monitoring large-scale gene expressions simultaneously have revealed mutual and highly correlated behaviors [18], [27]–[28]. This is conceivable due to the fact that gene expressions, i.e., net mRNA concentrations, are tightly controlled by the transcriptional system (consisting of transcription factors, RNA degradation, DNA physiochemical modifications, etc.) which regulates multiple sets of genes rather than a single gene. Hence, the use of correlation metrics has been widely adopted in microarray studies.

The majority of studies have used Pearson correlation, *r*(***X,Y***), when analyzing two *N*-dimensional expression vectors, ***X*** and ***Y***, e.g., comparing the response of genomes between two time points for a given biological stimulation;
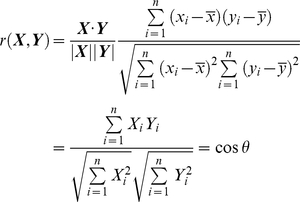
(1)where 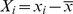 and 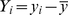, 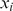 and 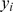 are gene expression of the *i^t^* ^h^ gene of expression vectors, ***X*** and ***Y***, respectively, 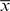 and 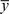 are mean values, and *θ* is the angle between the two *N*-dimensional expression vectors from the center of mass. Thus, this form of analysis of gene expressions reveals linear relationship, e.g., *θ* = 0 for perfect positive linear relationship, *θ* = π for perfect negative (anti-correlated) linear relationship and *θ* = π/2 for linearly independent relationship.

However, if the relationship between the two vectors is non-linear, then Pearson correlation analysis is insufficient. In such cases, the use of mutual information has been instrumental in biology [29]–[30]. In this paper, we adopted both a modified version of Pearson correlation (see below) and mutual information, to investigate the whole genome collective dynamics in the process of neutrophil differentiation to two distinct stimuli (atRA and DMSO), revealing the existence of neutrophil attractor as well as the whole genome expression dynamics toward the attractor.

### Modified Pearson correlation r_v_ for measuring expression variation

Each stimulus's dynamic genome expression activity (partial and whole) is defined by *N*-dimensional gene deviation-from-average vectors at time *t_i_* (*i* = 0,1,…,*M*), 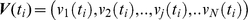, where 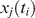 is expression value of the *j* ^th^ gene at *t_i_*, 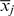 is its average expression over *M*+1 discrete time points, and 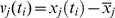 is its deviation from the average gene expression (called gene deviation). In our study, *N* = 12625 genes/ORFs and *M* = 12, where *t* = 0, 2, 4, 8, 12, 18, 24, 48, 72, 96, 120, 144, 168h. We modified the typical Pearson *r* (see Eq.1) to be 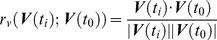 (see Figure S3 for *r_v_* vs. *r*) by subtracting their average expression value, 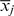, from each expression value at all time points, instead of subtracting the mean of whole genome expression, 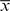. This index thus measures the temporal correlation of genome-wide expression deviations from their average values so as to allow discriminating gene expressions with different amplification but similar temporal profiles. For simplicity we included ORFs as genes, and for the microarray data, we applied RMA normalization which is known to produce robust reproducible results for all range of expression units [31].

### Mutual information I

Nonlinear dependency between vectors ***V***(*t_i_*) and ***V***(*t*_0_) is checked by mutual information [32]–[33] 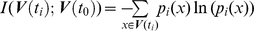 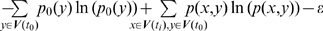, where the joint probability distribution function *p*(*x,y*), and marginal probability distribution functions, *p_i_*(*x*) at *t_i_* and *p*_0_(*y*) at *t_0_* are estimated by means of an histogram-based approach by discretizing the gene expression into *K* = 10 bins [32]. Note: due to the discretization, mutual information *I* incurs a systematic error *ε* [32]. Since randomly ordered data should destroy correlations, we expect *I* to be close to zero, therefore, we calculated the minimum *I* for 100 random permutations of gene deviation vectors {***V***(*t_i_*)}. However, we found a positive value for minimum *I* instead of zero, and so subtracted this minimum positive constant value from the final *I*. For comparing *I* of atRA and DMSO response, we used the normalized 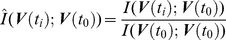 and called 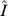 as *I* throughout the text.

### Ranking gene ensembles

The whole genome deviation vector at *t_0_*, ***V***(*t_0_*) = (*v_1_*(*t_0_*),*v_2_*(*t_0_*),..,*v_N_*(*t_0_*)) (*N* = 12625) was sorted according to the standard deviation, 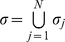, from the highest to the lowest, where 
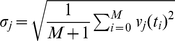
 (*M* = 12). The resultant ranked whole genome vector at *t_0_* is represented by 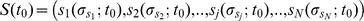, where 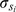
<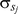 for *i*>*j* and 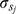 is the standard deviation of the *j^th^* gene deviation.

Next, we split the whole genome into *p* groups (each having *n* genes) at *t_0_*, so that the whole genome at *t_0_* is represented by 
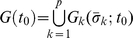
,where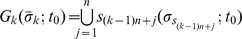, 
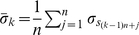
, 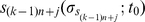 is the *j^th^* gene deviation in the *k^th^* group, and 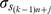 is its standard deviation. Note that we choose *p* to be an integer value of *N*/*n* for *n* = 10, 50, 100, 200, 500, 1000, and the residual genes were not evaluated. From here onwards, we simplified all notations without *σ* symbols, e.g., 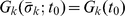. The set of *p* groups' average gene deviation from the whole genome at *t_i_* is represented by 
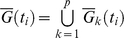
, where 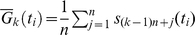 and *i* = 0,1,..,12.

## Supporting Information

Figure S1 Comparing CV with σ. Standard deviation, σ versus CV for genome elements of *n_t_* = 200 genes sorted by σ.
(0.18 MB TIF)

Figure S2 Identifying genome elements that form genome vehicles. (A) Schematic of iterative procedure to exhaustively determine genome elements falling into attractor (i.e., the genome vehicle, see maintext). (B) Number of genome elements falling into the attractor with respect to the number of iterations. We terminated the iteration procedure until the 4 consequential iterations do not constitute any genome element falling into the attractor. Since the number of gene shift other than 50 is not sensitive to the characteristics of the genome vehicle, we chose 50 genes shift to save the computational time. We obtained a total of 21 and 28 genome elements constituting the genome vehicles for atRA for DMSO, respectively.
(0.56 MB TIF)

Figure S3 Relationship between *r* and *r_v_*. *r* and *r_v_* are obtained for *n* = 200 randomly selected genes with 3000 repeats (each represented by a dot) from the entire data containing 13 time points (*i* = 0,…,12).
(0.30 MB TIF)
