# Supplementary figures and images for: Collective Dynamics of Specific Gene Ensembles Crucial for Neutrophil Differentiation: The Existence of Genome Vehicles Revealed
Source: PLoS One. 2010 Aug 11;5(8):e12116. doi: 10.1371/journal.pone.0012116 (PMC2920325; doi:10.1371/journal.pone.0012116)

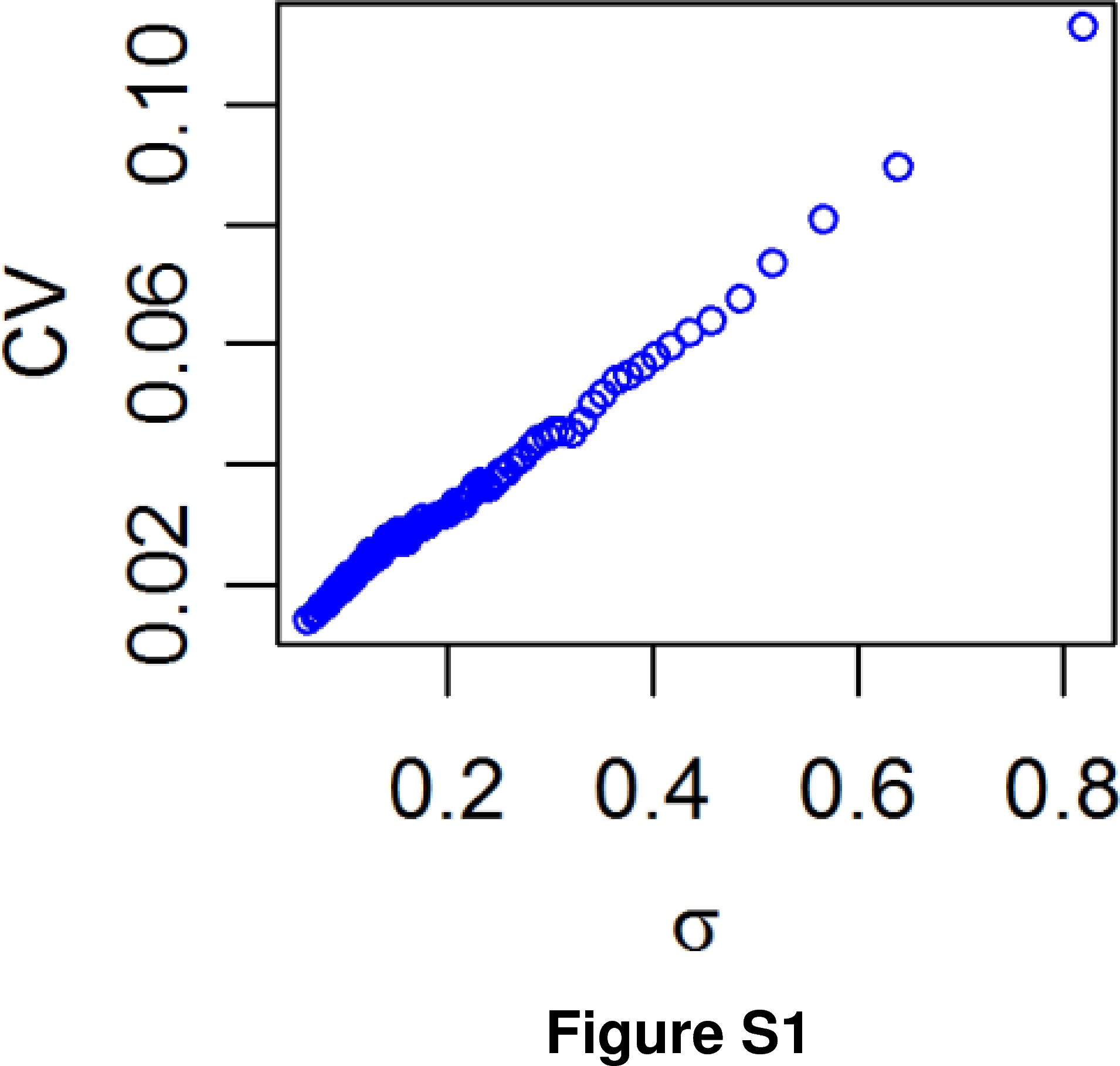

Supplement: Figure S1 — Comparing CV with σ. Standard deviation, σ versus CV for genome elements of nt = 200 genes sorted by σ. (0.18 MB TIF) [file pone.0012116.s001.tif]

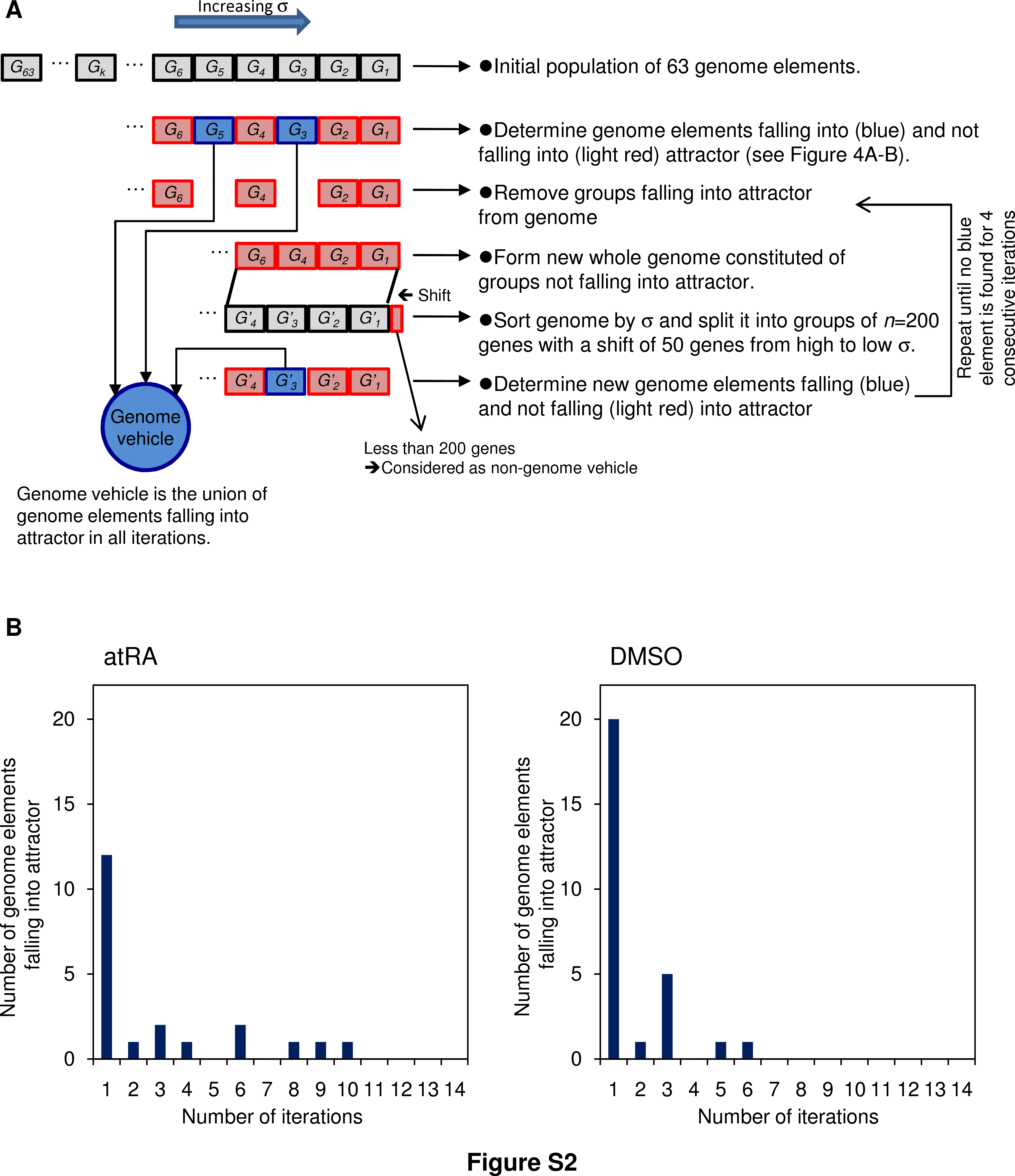

Supplement: Figure S2 — Identifying genome elements that form genome vehicles. (A) Schematic of iterative procedure to exhaustively determine genome elements falling into attractor (i.e., the genome vehicle, see maintext). (B) Number of genome elements falling into the attractor with respect to the number of iterations. We terminated the iteration procedure until the 4 consequential iterations do not constitute any genome element falling into the attractor. Since the number of gene shift other than 50 is not sensitive to the characteristics of the genome vehicle, we chose 50 genes shift to save the computational time. We obtained a total of 21 and 28 genome elements constituting the genome vehicles for atRA for DMSO, respectively. (0.56 MB TIF) [file pone.0012116.s002.tif]

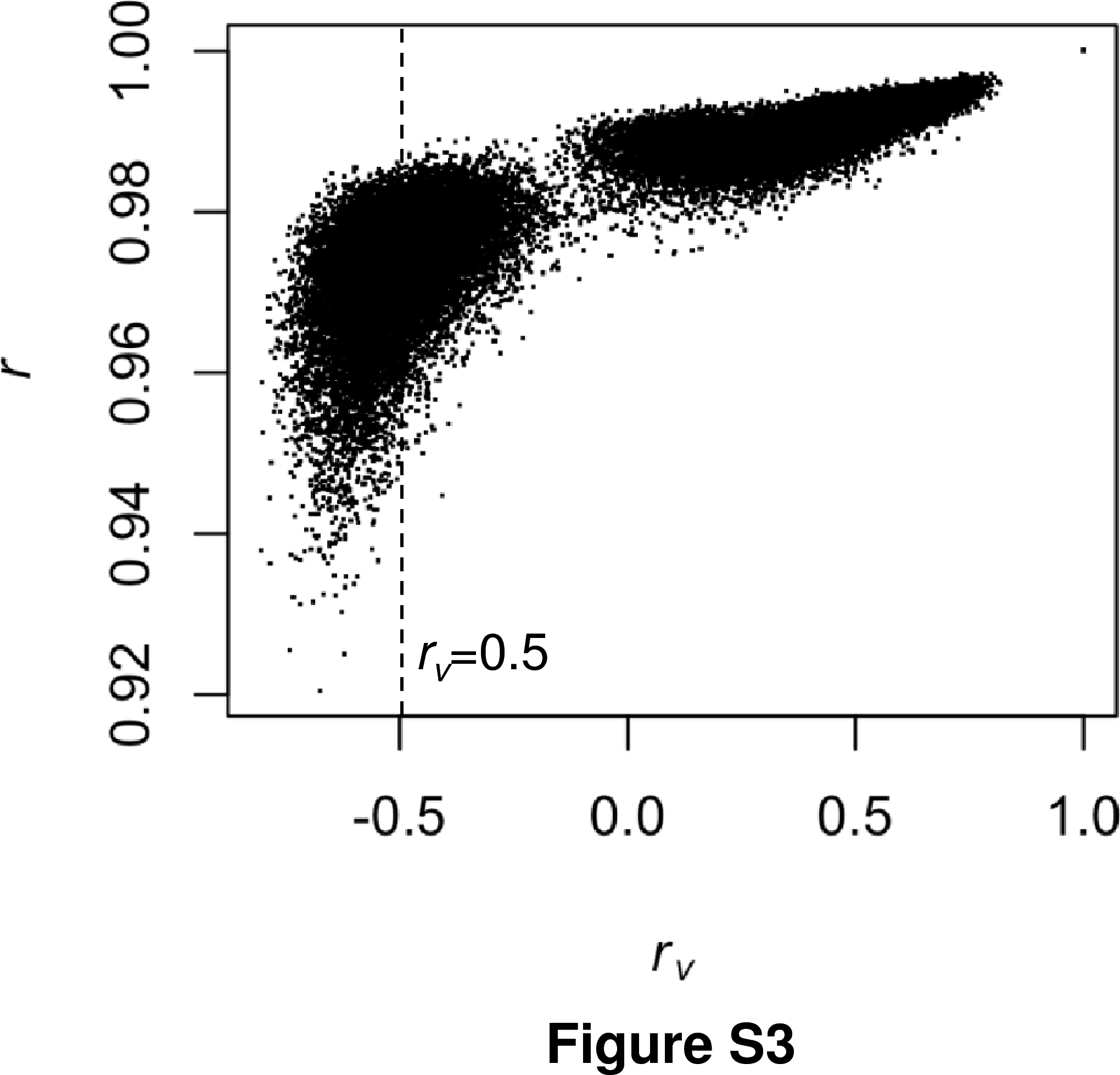

Supplement: Figure S3 — Relationship between r and rv. r and rv are obtained for n = 200 randomly selected genes with 3000 repeats (each represented by a dot) from the entire data containing 13 time points (i = 0,…,12). (0.30 MB TIF) [file pone.0012116.s003.tif]
